# Flow cytometric analysis of inflammatory and resident myeloid populations in mouse ocular inflammatory models

**DOI:** 10.1016/j.exer.2016.08.007

**Published:** 2016-10

**Authors:** Sidath E. Liyanage, Peter J. Gardner, Joana Ribeiro, Enrico Cristante, Robert D. Sampson, Ulrich F.O. Luhmann, Robin R. Ali, James W. Bainbridge

**Affiliations:** a UCL Institute of Ophthalmology, 11-43 Bath Street, London, EC1V 9EL, UK; b NIHR Biomedical Research Centre for Ophthalmology, Moorfields Eye Hospital and UCL Institute of Ophthalmology, City Road, London, EC1V 2PD, UK

**Keywords:** Myeloid cells, Retinal microglia, Neutrophils, Monocytes, Flow cytometry, Experimental autoimmune uveoretinitis, Endotoxin-induced uveitis, Choroidal neovascularization, DC, dendritic cell, EAU, experimental autoimmune uveitis, FACS, Fluorescence-activated cell sorting, EIU, Endotoxin-induced uveitis, CNV, Choroidal neovascularization, PBS, Phosphate buffered saline, CCR2, C-C chemokine receptor 2, RPE, Retinal pigmented epithelium

## Abstract

Myeloid cells make a pivotal contribution to tissue homeostasis during inflammation. Both tissue-specific resident populations and infiltrating myeloid cells can cause tissue injury through aberrant activation and/or dysregulated activity. Reliable identification and quantification of myeloid cells within diseased tissues is important to understand pathological inflammatory processes. Flow cytometry is a valuable technique for leukocyte analysis, but a standardized flow cytometric method for myeloid cell populations in the eye is lacking. Here, we validate a reproducible flow cytometry gating approach to characterize myeloid cells in several commonly used models of ocular inflammation. We profile and quantify myeloid subsets across these models, and highlight the value of this strategy in identifying phenotypic differences using *Ccr2*-deficient mice. This method will aid standardization in the field and facilitate future investigations into the roles of myeloid cells during ocular inflammation.

## 1 Introduction

Myeloid cells and their contribution to tissue homeostasis and inflammation represent a key area in human immunological disease. Myeloid cells have been historically divided into resident and infiltrating populations. Depending on the inflammatory stimuli, each of these populations can exhibit a wide range of actions and roles such as driving acute inflammatory responses and both initiating and regulating lymphocyte-driven adaptive responses (Gordon et al., 2014, Janeway and Medzhitov, 2002). Resident myeloid cells continuously sample the tissue micro-environment acting to trigger inflammatory responses to pathogens and tissue trauma and pave the way for the progressive recruitment of circulating myeloid cells such as neutrophils and inflammatory monocytes. Importantly, both resident and infiltrating myeloid cells can themselves cause tissue injury through their aberrant activation and/or excessive dysregulated activity (Murray and Wynn, 2011).

Progress in understanding myeloid cell biology has been aided by technological advances in flow cytometry that allow staining of more than 17 individual antibody-fluorophore combinations for analysis (Perfetto et al., 2004), facilitating in-depth immunophenotyping of myeloid cells and their contribution to disease. Careful validation of this powerful technique, across specific tissues and cell types for the markers used, is critically important for reliable interpretation and meaningful comparison. There is currently no consensus regarding a standardized flow cytometry panel for myeloid cells, in part due to the lack of definitive myeloid cell lineage markers (Hume, 2011). This has resulted in widespread variation in the use of cell surface markers to define myeloid cell populations (Murray et al., 2014).

The inconsistent identification of myeloid cells hinders standardization in the field, and deleteriously affects reproducibility, interpretation and comparison of results generated from different multi-color panels. Investigators of gene expression in the mouse immune system recognized this problem and proposed a standardized approach to fluorescence-activated cell sorting (FACS) of single leukocyte sub-populations (Gautier et al., 2012). However, these strategies are unsuitable for assessing multiple infiltrating myeloid cell populations within a tissue. To this end, Rose et al. proposed a novel gating strategy for analyzing the splenic myeloid compartment (Rose et al., 2011).

The same considerations apply to characterization of myeloid populations in the eye during inflammation. Traditionally involved in leukocyte-driven inflammatory diseases, myeloid cells have now been implicated in chronic progressive eye conditions such as age-related macular degeneration (AMD) (Bainbridge, 2007, Luhmann and Ali, 2011). Neutrophils mediate tissue damage through production of reactive oxygen species (ROS) and inflammatory monocytes contribute both to disease progression and regulation of inflammation during models of uveitis (Kerr et al., 2008, London et al., 2013). Additionally, pro-angiogenic factors produced in response to macrophages contribute to angiogenesis during laser-induced choroidal neovascularization in mice (Sakurai et al., 2003). While CD11b^+^ myeloid cell subsets in these models have been described using flow cytometry, CD11b is not exclusively myeloid-expressed, and resident CD11b^+^ microglia, dendritic cells and perivascular macrophages are difficult to differentiate as population phenotyping is inconsistent and unvalidated.

We apply the strategy reported by Rose et al. to describe a detailed and validated approach to the analysis of myeloid cell populations in the retina and retinal pigmented epithelium (RPE)-choroid complex during inflammation. This strategy employs specific antibody clones targeting the cell surface proteins CD11b, CD11c, Ly6G, Ly6C, NK1.1 in combination with the FSC and SSC parameters and a live/dead cell discrimination dye. Whilst positivity for CD11c, Ly6G and NK1.1 discriminates dendritic cells, neutrophils and NK cells respectively, circulating monocytes can be characterized into two populations using cell surface expression of Ly6C. Ly6C^+^ cells are rapidly recruited to sites of inflammation and Ly6C^neg^ cells patrol blood vessels and in the tissue comprise resident microglia (Auffray et al., 2009).

Using *Ccr2*^*−/−*^ knockout mice that exhibit impaired recruitment of myeloid cells to sites of inflammation (Newson et al., 2014), we show that this strategy can reveal phenotypic differences between myeloid populations in laser-induced CNV. We highlight and address specific issues of this gating strategy with regards to its application to ocular tissue to provide an additional resource for the investigation of ocular biology.

## 2 Materials and supplies

### 2.1 Animals

All in vivo procedures were conducted under the regulation of the UK Home Office Animals (Scientific Procedures) Act 1986, and the study was in compliance with the ARVO Statement for the Use of Animals in Ophthalmology and Vision Research. C57BL/6J and *CCR2*^*−/−*^ (C57BL/6J background) mice were used in this study. All mice used in this study were 6–10 weeks old at the time of procedure.

### 2.2 Buffers

Flow buffer consisted of PBS with 2 mM EDTA and 0.1% (w/v) bovine serum albumin (all Sigma-Aldrich Ltd., UK). Sorting buffer comprised DMEM, 2% (v/v) fetal calf serum, 10 mM HEPES (all Invitrogen; Life Technologies Limited, UK).

## 3 Detailed methods

### 3.1 Induction of experimental autoimmune uveoretinitis (EAU)

For the induction of EAU in C57BL/6J mice, human RBP 1–20 peptide (H-Gly-Pro-Thr-His-Leu-Phe-Gln-Pro-Ser-Leu-Val-Leu-Asp-Met-Ala-Lys-Val-Leu-Leu-Asp-OH, 10 mg/ml; Insight Biotechnology, UK) was mixed with an equal volume of Complete Freund's Adjuvant (Sigma Aldrich, UK) supplemented with 1.5 mg/ml of Mycobacterium tuberculosis H37R (DIFCO Laboratories, UK) to create an emulsion. 50 μl was subcutaneously injected into each flank to give a total dose of 500 μg per mouse. 1.5 μg of Pertussis toxin (Tocris Bioscience, UK) was then administered intraperitoneally (Cortes et al., 2008).

### 3.2 Induction of endotoxin-induced uveitis (EIU)

EIU was induced by intravitreal administration of 1 ng of lipopolysaccharide (Sigma Aldrich, UK) into each eye using a microsurgical syringe and 38 gauge needle (Hamilton, Switzerland) (Trittibach et al., 2008).

### 3.3 Laser-induced choroidal neovascularization

The fundus was visualized by coupling a microscope coverslip (VWR International Ltd., UK) to the ocular surface using a viscous topical ocular lubricant (Viscotears; Novartis, UK). Choroidal neovascularization was induced by rupturing Bruch's membrane using a slit-lamp-mounted diode (680 nm) laser system (Keeler, UK) with the following settings: 210 mW power; 100 ms duration; 100 μm spot diameter. These settings consistently generate a subretinal gas bubble indicative of successful rupture of Bruch's membrane and consequent induction of choroidal neovascularization (Balaggan et al., 2006). Ten burns were delivered to ensure sufficient cell numbers for flow cytometric analysis and FACS.

### 3.4 Preparation of cell suspension

Eyes were dissected microsurgically in 1 ml ice-cold RPMI 1640 (Invitrogen; Life Technologies Limited, UK) to isolate the retina (excluding the ciliary marginal zone) and RPE-choroid complex. For laser-induced CNV, the retinas and RPE-choroid complexes of both eyes were pooled for each animal before undergoing enzymatic digestion with a solution containing 25 mg/ml DNAse (Sigma-Aldrich Limited, UK) and 10 mg/ml Collagenase D (Roche Diagnostics, UK) for 30 min at 37 °C. The digested material was homogenised by gentle trituration with a pipette, before being filtered. For EIU and EAU, the dissected retina was transferred into a 1.5 ml reaction tube and mechanically dissociated by rapping the tube across a reaction tube rack ten times, before being filtered. Filtration was accomplished by passing the sample through a 60 μm mesh filter plate (Millipore UK Ltd., UK) by centrifugation (300 g for 5 min). The supernatant was discarded and the sample resuspended with either flow or sorting buffer.

Splenic samples were prepared by homogenising the tissue between two frosted polysine slides (VWR International Ltd., UK) in 1 ml ice-cold phosphate buffered saline (PBS; Sigma-Aldrich Limited, UK). Samples were centrifuged (300 g for 5 min) and the cell pellet underwent red blood cell lysis using 1 ml ACK buffer (Invitrogen; Life Technologies Limited, UK) for 20 s. Lysis was terminated by adding 50 ml PBS to the sample, and the sample was filtered with a 70 μm cell strainer (BD Labware, UK) and centrifuged (300 g for 5 min). The resultant cell pellet was resuspended with either flow or sorting buffer.

### 3.5 Surface marker staining for flow cytometry

The cell filtrate was suspended in ice-cold flow buffer to produce a single cell suspension and blocked with rat anti-mouse CD16/32 clone 2.4G2 Fc block (5 ng/ul; BD Biosciences, UK) for 10 min at 4 °C. Surface staining was performed in the dark for 20 min at 4 °C in flow buffer. Cells were washed between each incubation with flow buffer. Intracellular staining was performed after fixation and permeabilization (BD Cytofix/Cytoperm; BD, UK).

A list of titrated surface markers for these experiments includes: CD4 clone GK1.5 PE (2 ng/ul; BioLegend., UK); CD8a 53–6.7 PE (2 ng/ul; eBioscience Ltd., UK); CD8 clone 53–6.7 APC (2 ng/ul; BD Biosciences, UK); B220 RA3-6B2 PE (2 ng/ul; eBioscience Ltd., UK); CD11b clone M1/70 PE-Cy7 (2 ng/ul; eBioscience Ltd., UK); CD11b clone M1/70 BV711 (0.5 μg/μl; Biolegend Ltd., UK), CD11c clone N418 FITC (1.25 ng/ul; eBioscience Ltd., UK); Ly6G clone 1A8 APC (2 ng/ul; eBioscience Ltd., UK); Ly6G clone 1A8 BV421 (1.25 ng/ul; Biolegend Ltd., UK); NK1.1 clone PK136 PerCP/Cy5.5 (2.5 ng/ul; Biolegend, Ltd., UK); NK1.1 clone PK136 APC (2 ng/ul; eBioscience Ltd., UK); Ly6C clone HK1.4 BV510 (0.63 ng/ul; Biolegend, Ltd., UK); F4/80 clone CI:A3-1 RPE (AbD Serotec, UK); live-dead discrimination with SYTOX Blue (2.5 μM/μl; Invitrogen; Life Technologies Limited, UK) or DRAQ7 (1.5 μM/μl; Biostatus Ltd., UK). Intracellular staining for IBA-1 was performed using an antibody obtained by conjugating a rabbit anti-Iba-1 antibody (Wako GmbH, Germany) with AlexaFluor 647 (Molecular Probes antibody labelling kit; Life Technologies Limited, UK).

### 3.6 Data acquisition and fluorescence-activated cell sorting (FACS)

Fluorescence-minus-one controls were used for gating analyses to distinguish positively from negatively staining cell populations. Compensation was performed using single color controls prepared from BD Comp Beads (BD Biosciences, UK) for cell surface staining or dead splenocytes (heated at 65 °C for 1 min) for live-dead discrimination. Flow cytometric analysis was carried out with a Fortessa X-20 (BD Biosciences, USA) equipped with 355 nm, 405 nm, 488 nm, 561 nm and 640 nm excitation lasers. All samples were run at 6 μl/minute (low setting) and data analysis was performed with FlowJo v.10.0.7 (FlowJo LLC, USA). FACS was performed using an Influx (BD Biosciences, USA) equipped with 355 nm, 405 nm, 488 nm, 561 nm and 640 nm excitation lasers. All data collection and sorting were performed using BD FACS Diva software (BD Biosciences, UK). Cells were sorted into DMEM media containing 50% FCS (Invitrogen; Life Technologies Limited, UK) using 50 psi and a 70 μm nozzle.

Absolute cell numbers were obtained by using a standard curve constructed using serial dilutions of 5 × 10^4^ Accucount blank particles (Spherotech, Germany) in a set volume of 300 μl; each dilution was run at 6 μl/min for 3 min. Before acquisition, retinal and RPE-choroidal samples were resuspended in 300 μl and run at 6 μl/min for 3 min. The identical volumes and acquisition rates allowed interpolation of absolute cell numbers using Graphpad Prism v6 (Graphpad Software Inc., USA).

### 3.7 Cytospin preparation and staining

For cytologic analysis of cell preparations, cells were mounted on slides using a Cytospin centrifuge (ThermoElectron, UK) for 5 min at 1000 RPM. Cells were air-dried, then fixed and stained using a Romanowsky stain (Shandon Kwik-Diff; (Fisher Scientific, UK)) according to manufacturer's instructions. Cytospin preparations were imaged using a standard light microscope using a 40× (Diaplan; Leitz, Germany). Independent analysis of the slides was performed by an accredited cytologist masked to the study who assessed homogeneity of the cytopsin preparation over 20 fields of view.

### 3.8 Immunohistochemistry

Retinal flat mounts were prepared as described before (Luhmann et al., 2012). Briefly, eyes were removed and fixed at room temperature for 5 min in 4% buffered paraformaldehyde (PFA); retinas were dissected and post-fixed at room temperature in 4% PFA 10 min. Tissue samples were washed twice with PBS, blocked in blocking solution (1% (w/v) BSA (Sigma Aldrich, UK), 5% (v/v) nonspecific goat serum (AbD Serotec, UK), 1% (v/v) Triton X-100 (Sigma Aldrich, UK)) for 1 h at room temperature. Samples were incubated over-night at 4 °C with the following primary antibodies, diluted in blocking solution: rabbit anti-Iba1 clone 019–19741 (1:500, Wako GmbH, Germany), rat anti-Ly6C clone HK1.4 (1:200, Abcam, UK). The next day, after washing 3 × 10 min with blocking solution, flat-mounts were incubated for 2 h at room temperature with AlexaFluor 488-conjugated anti-rabbit IgG and AlexaFluor 546-conjugated anti-rat IgG secondary antibodies (1:500; Molecular Probes; Life Technologies Ltd., UK) in blocking buffer. Samples were washed 3 × 10 min with blocking buffer, 1 × 10 min with PBS and mounted on a glass slide with fluorescence mounting medium (Dako Fluorescence Mounting Medium; Dako, UK). Flat-mount tissue was examined by confocal laser scanning microscopy (Leica DM5500Q; Leica Microsystems, Germany), using a 40× objective. High-resolution z-stack images (0.5 μm thickness) of regions of interest were acquired and z-projected to generate the images presented.

### 3.9 Statistical analysis

Data were plotted and analysed for statistical significance in Graphpad Prism v6 (Graphpad Software Inc., USA) using the non-parametric Mann Whitney (two-tailed) method. P-values of ≤0.05 were considered significant.

### 3.10 Gating strategy is reproducible in the splenic myeloid compartment

We reproduced and cytologically validated the gating strategy published by Rose et al. using different antibody-fluorophore combinations (Rose et al., 2011). Following exclusion of debris and cellular aggregates, a live/dead discrimination was performed. Plotting CD11b against CD11c allowed dendritic cells (CD11c^high^CD11b^+^) to be differentiated from other CD11b^+^ myeloid subsets. The latter cell population was then analysed by plotting NK1.1 against Ly6G. NK1.1 is a marker for natural killer cells which are thought to be lymphoid cells deriving predominantly from lymphoid progenitors (Vivier et al., 2008, Yu et al., 2013) but can constitute a large proportion of CD11b^+^ cells.

**Fig. 1:**
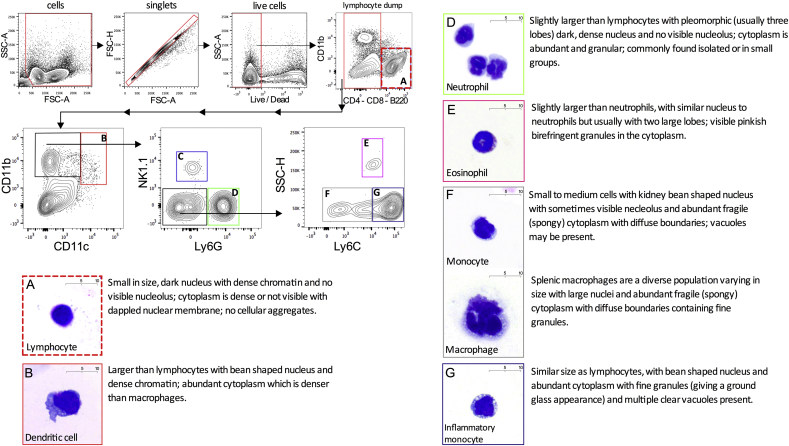
**Identification and cytology validation of myeloid leukocytes in the mouse spleen using an established flow cytometry gating strategy**. The flow cytometry gating strategy shows sequential exclusion of debris, doublets and dead cells from total C57BL/6 mouse splenocytes. Lymphocytes were excluded in a dump gate using CD4, CD8 and B220 in the same channel (gate A). CD11b^+^CD11c^high^ dendritic cells were then gated (gate B) followed by exclusion of NK1.1^+^ natural killer cells (gate C) and Ly6G^+^ neutrophils (gate D) The remaining cells were classified into eosinophils (gate E), resident (gate F) and inflammatory monocyte/macrophage populations (gate G) using side scatter (SSC) and Ly6C. Cytospin preparations from FACS sorted populations were prepared and stained with Romanowsky stain variant. These are shown with the corresponding flow cytometry gates (A–G) and cytology description. NK cells (gate C) have been excluded from cytological analysis as they are not classified as myeloid cells; n = 2 animals; 2 independent experiments.

The myeloid gating strategy allowed natural killer cells (CD11b^+^NK1.1^+^) and neutrophils (CD11b^+^Ly6G^+^) to be distinguished from the remaining monocytes and macrophages (CD11b^+^NK1.1^neg^Ly6G^neg^). These monocytes and macrophages can be classified into inflammatory (Ly6C^high^) and non-inflammatory or resident (Ly6C^low/neg^) based on the expression of the Ly6C antigen (Geissmann et al., 2010). FACS using this panel and strategy allowed each myeloid cell population to be isolated. Cells were identified using cytological characteristics based on nuclear morphology, nuclear/cytoplasmic ratio, cytoplasmic staining and granularity. Following fixation and Romanowsky staining, masked analysis by an accredited cytologist confirmed that the gating strategy isolated the intended myeloid cell populations in the spleen (Fig. 1).

### 3.11 Gating strategy applied to naïve retina and choroid

**Fig. 2:**
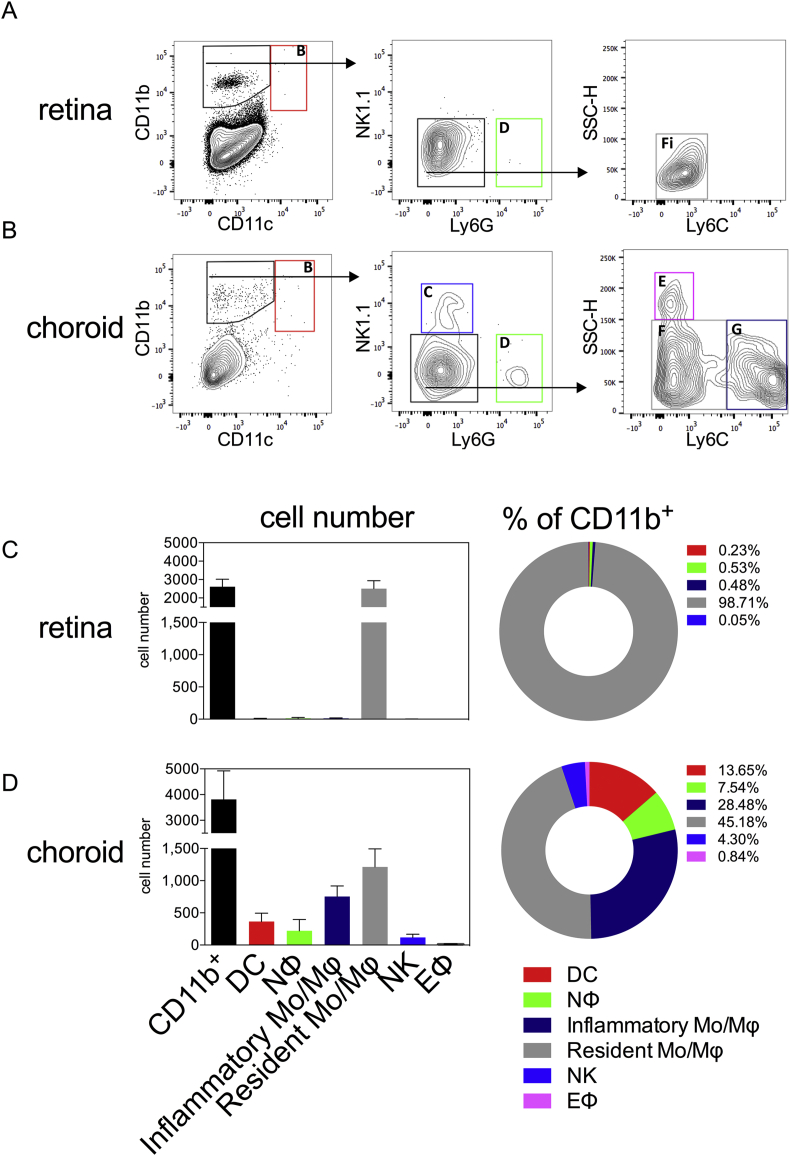
**Application of the myeloid gating strategy in the naïve mouse eye**. Analysis of the retinal (A) and RPE-choroidal (B) compartment with the gating strategy, representing the acquisition of single cell suspensions from either pooled retina or RPE-choroid from 1 animal. Absolute cell numbers and percentages of myeloid subsets present in the retina (C) and RPE-choroid (D) are shown. Gates are as follows: A – lymphocytes; B – dendritic cells; C – natural killer cells; D – neutrophils; E – eosinophils; F – resident monocyte/macrophages; G – inflammatory monocyte/macrophages; n = 4 animals (pooled eyes); mean ± SD shown; 2 independent experiments.

Once validated this gating strategy was applied to both naïve retina and RPE-choroid of wild type mice (Fig. 2A,B). In both tissues, there were low numbers of CD11b^+^ myeloid cells present (means of 2600 in the retina and 3800 in the RPE-choroid) (Fig. 2C,D). CD11b^+^CD11c^high^ classical dendritic cells were rare in retina compared to the RPE-choroid, consisting of 0.23% and 13.65% of the CD11b^+^ myeloid cell population respectively. Both neutrophils and inflammatory monocytes/macrophages were present in normal RPE-choroid, comprising 7.54% and 28.48% of the native CD11b^+^ myeloid population (Fig. 2D), with the remainder mainly composed of resident monocytes/macrophages (45.18%) with very small NK (4.30%) and eosinophil (0.84%) populations present. In normal retina, the lack of other inflammatory subsets, such as neutrophils and inflammatory monocytes/macrophages, was consistent with the absence of inflammatory stimuli in an immune privileged setting. Here, 98.71% of the CD11b^+^ myeloid cells present were resident monocytes/macrophages (CD11b^+^NK1.1^neg^Ly6G^neg^Ly6C^low/neg^) (Fig. 2C).

### 3.12 Retinal microglia identified as resident monocyte/macrophage population

In order to better understand the inflammatory processes involved, any gating strategy for models of ocular inflammation must accurately identify resident CD11b^+^ cells of which three populations are currently known, microglia, dendritic cells (DC) and perivascular macrophages (Chen and Xu, 2015). Studies with transgenic reporter mice have shown that the greatest in number of the three populations are retinal microglia (Kezic et al., 2008, London et al., 2013) and they serve to maintain tissue homeostasis (Dick, 2003). Lineage studies have shown that these tissue-resident macrophages are derived from the yolk sac, independent from haematopoiesis (Schulz et al., 2012). Parabiosis experiments have shown that these specialized cells self-maintain, remaining independent of contributions from circulating monocytes (Ajami et al., 2011, Hashimoto et al., 2013).

Microglia and DCs are CD11b^+^CD45^low^ and can be distinguished in steady state by DC expression of CD11c. The classical immunohistochemical marker for CNS microglia is the intracellular ionized calcium binding adapter molecule 1 (Iba-1), although it should be noted that Iba-1 is not myeloid-specific outside of the brain and retina (Imai et al., 1996). Staining for Iba-1 requires fixation and permeabilization and the morphological changes (from highly branched and ramified to ameboid) of microglia associated with their activation cannot be distinguished by flow cytometry or cytospin. Other groups have used the CD11b^+^CD45^low^ surface marker phenotype to define microglia as the dominant resident myeloid population in the retina (Kettenmann et al., 2011, Zhang et al., 2002). Perivascular macrophages, another resident myeloid population, expresses high levels of CD45, necessitating careful discrimination between low and high expression of CD45 (depending upon flourochrome choice and staining conditions) (Patel et al., 2013). The identification of microglia by the CD45^low^ phenotype is compounded by results in models of CNS disease that show that microglia can upregulate surface expression of CD45 (Ponomarev et al., 2005).

We sought to examine the outlined gating strategy in steady state conditions and so analysed the retina and RPE-choroid from naïve mice (Fig. 2A and B). Resident monocytes/macrophages (CD11b^+^NK1.1^neg^Ly6G^neg^Ly6C^low/neg^) formed the majority of CD11b^+^ myeloid cells in the normal wild type retina (Fig. 2C). In the RPE-choroid complex resident monocytes/macrophages were the dominant population although we could detect low cell counts from all other myeloid subtypes identified using the gating strategy. This tissue is highly vascularized and the presence of these other subsets in steady state could represent the detection of circulating myeloid cells normally present in systemic circulation.

**Fig. 3:**
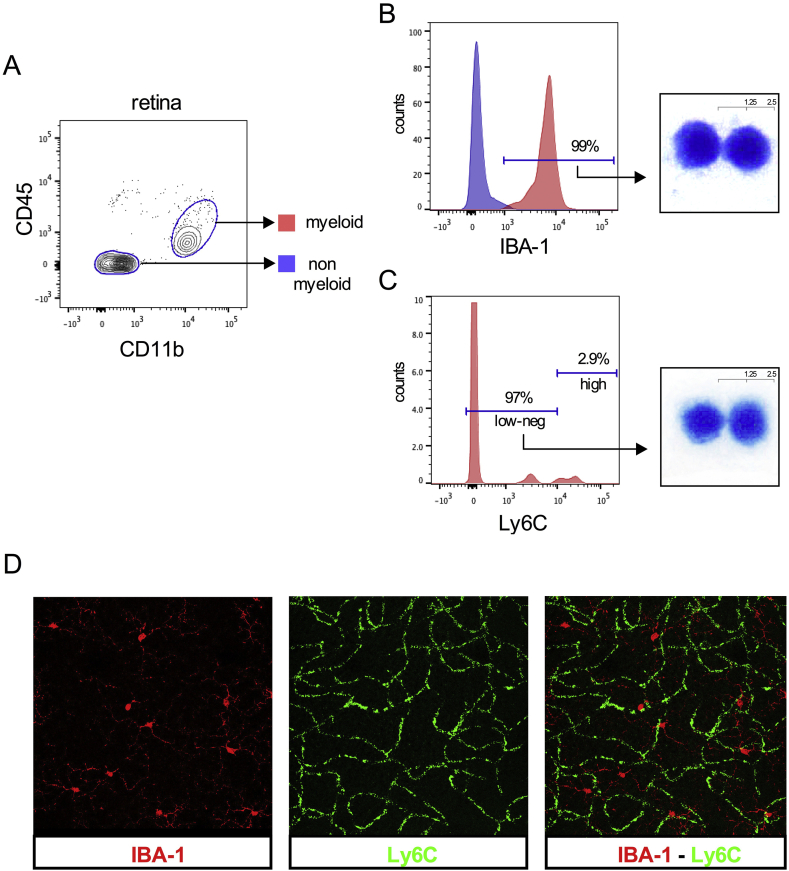
**Confirmation of the resident monocyte/macrophage population identified in the naïve retina as retinal microglia**. (A) The myeloid cell population in naïve retina was defined by the CD11b^+^CD45^low^ immunophenotype. (B) 99% of this CD11b^+^CD45^low^ population was positive for the intracellular microglia marker IBA-1. (C) The majority (97%) of the CD11b^+^CD45^low^ population is Ly6C^low/neg^. (D) Confocal microscopy of retinal flatmount showing IBA-1 (red) and Ly6C (green) staining. A composite image reveals no co-localization between IBA-1 and Ly6C suggesting that the majority of the Ly6C stain in this immunohistochemistry originates from endothelial cells; n = 3 animals (individual eyes); 3 independent experiments.

To further confirm our assumption that the resident monocyte/macrophage population in this gating strategy corresponds to retinal microglia in steady state, we analysed the retinal microglia (CD11b^+^CD45^low^) subset in naïve retina for Iba-1- and Ly6C-positivity (Fig. 3A). These data revealed that, consistent with previous studies, the majority of the CD11b^+^CD45^low^ cell population was Iba-1^+^ retinal microglia cells (Fig. 3B). The preponderance of Iba-1^+^ retinal microglial cells was Ly6C^low/neg^, confirming that the CD11b^+^Ly6C^low/neg^ subset in the gating strategy was composed mainly of retinal microglia (Fig. 3C). FACS sorting of CD11b^+^CD45^low^Iba-1^+^ and CD11b^+^Ly6C^low/neg^ populations from naïve retina revealed that the masked cytological analysis of the individual populations showed no difference between the nuclear morphology of these populations (Fig. 3B, C); assessment of cytoplasmic features was not possible as the permeabilization step necessary for intracellular Iba-1 staining caused cytoplasmic rupture and loss in subsequent processing.

As a further means of validation, immunohistochemistry was performed using Iba-1 and Ly6C on retinal flatmounts of naïve eyes (Fig. 3D). The majority of Ly6C expression was derived from endothelial cells (Jutila et al., 1988). Confocal microscopy highlighted the absence of co-localization between Iba-1 and Ly6C, providing immunohistochemical confirmation that Iba-1^+^ cells have a Ly6C^low/neg^ phenotype.

### 3.13 Using the *Rose* et al. flow cytometry gating strategy in different ocular inflammatory models

Following the validation of the *Rose* et al. flow cytometry gating strategy on naïve ocular tissue we applied it to three mouse models of intraocular inflammation: experimental autoimmune uveitis (EAU), endotoxin-induced uveitis (EIU), and laser-induced choroidal neovascularization (CNV), for evaluation of differential immune phenotyping.

EAU is an antigen-specific T cell-driven model of human pan-uveitis comprising infiltration of T cells and multiple myeloid cell populations (Gardner et al., 2015). EIU is a sterile LPS-driven self-resolving model of myeloid infiltration and migration into the eye mirroring aspects of human anterior uveitis (Trittibach et al., 2008). Retinal tissues were harvested at 18 h and 26 days post induction for EIU and EAU respectively, corresponding to peak levels of leukocyte infiltration (Chu et al., 2013, Trittibach et al., 2008). As the Bruchs membrane stays intact during EIU and EAU, the choroid is not typically analyzed in these models. Laser-induced CNV represents an acute tissue inflammation model that results in neovascularization, sharing similarities with wet age-related macular degeneration. Myeloid infiltration peaks 2–3 days post laser induction (Sakurai et al., 2003, Tsutsumi et al., 2003) and analysis of the infiltrate in retina and RPE-choroid was performed at day 3.

**Fig. 4:**
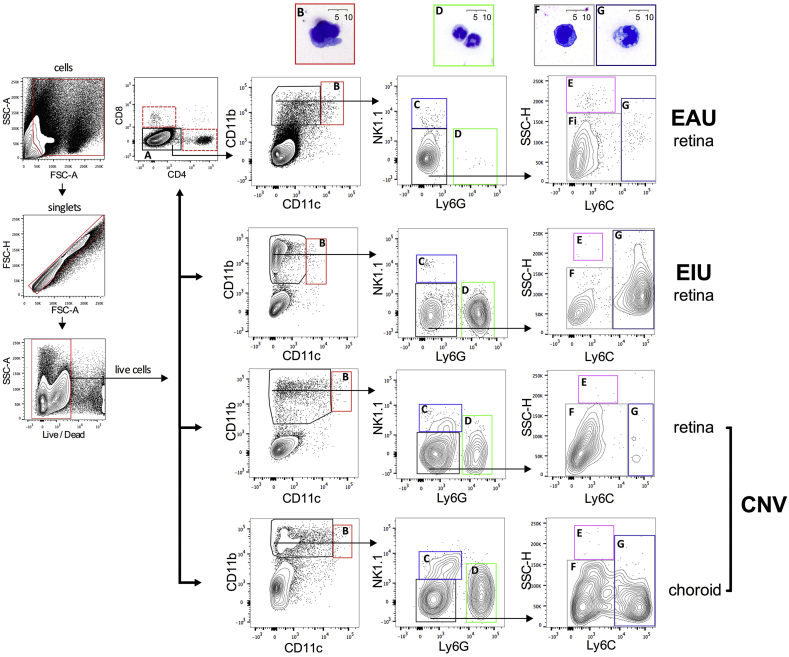
**The myeloid gating strategy distinguishes resident from infiltrating cells in mouse models of ocular inflammation**. Flow cytometry plots show the myeloid gating strategy as applied to dissected mouse eyes following induction of three different ocular inflammatory models, experimental autoimmune uveitis (EAU), endotoxin induced uveitis (EIU) and laser induced choroidal neovascularization (CNV). Cytospin preparations from FACS sorted populations (indicated by gate and color) during ocular inflammation are shown above the relevant plots. EAU and EIU plots are representative of data from individual eyes, while eyes were pooled per animal for CNV. Gates are as follows: A – lymphocytes; B – dendritic cells; C – natural killer cells; D – neutrophils; E – eosinophils; F – resident monocyte/macrophages; G – inflammatory monocyte/macrophages. Cytology from dendritic cells (gate B), neutrophils (gate D), resident monocyte/macrophages (gate F) and inflammatory monocyte/macrophages (gate G) are shown; n = 12 animals (pooled eyes); 2 independent experiments.

The flow cytometry gating strategy was reproducible in all three models with retina, and with RPE-choroid in laser-induced CNV (Fig. 4).

Masked cytological analysis of fixed and stained FACS-isolated populations confirmed that the cell populations corresponded to the splenic isolates and known cytological characteristics.

**Fig. 5:**
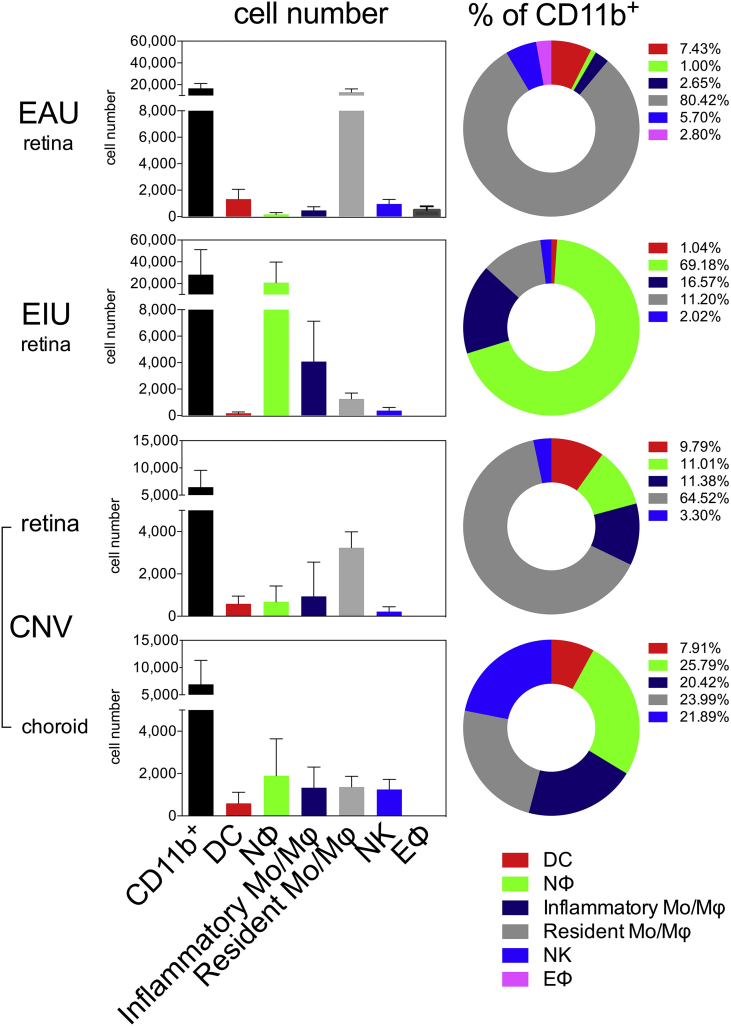
**Quantification of myeloid populations in the eye during mouse models of ocular inflammation**. Charts show the quantification of the absolute cell counts and percentages of gated subsets of the total CD11b^+^ population in the various models at peak disease; n = 6 animals (pooled eyes for CNV and EAU, individual eyes for EIU); mean ± SD shown; 2 independent experiments; colors in individual bars match individual population gate from Fig. 1, Fig. 4. Although not myeloid cells, CD11b^+^ NK cells are included as they constitute a significant proportion in EAU and CNV.

The data acquired using the gating strategy enabled us to compare peak disease across the different models both qualitatively and quantitatively. At peak disease in all models we observed elevated absolute counts of total CD11b^+^ myeloid cells (Fig. 5). This is due to greater infiltrating numbers of classical dendritic cells (CD11b^+^CD11c^high^), inflammatory monocyte/macrophages (CD11b^+^NK1.1^neg^Ly6G^neg^Ly6C^high^) and non-myeloid natural killer cells (CD11b^+^NK1.1^+^) into the eye. In EIU, the myeloid infiltrate into the retina was composed of mainly neutrophils (CD11b^+^Ly6G^+^) and inflammatory monocytes (CD11b^+^Ly6C^+^). Neutrophils were also detected in the retina and RPE-choroid at peak disease of laser-induced CNV and very low numbers were observed infiltrating the retina at peak EAU disease (Fig. 5). This low number of infiltrating neutrophils during peak EAU observed in C57BL/6 mice here reflects the mouse genetic background as neutrophils can form a significant component of the inflammatory infiltrate during EAU when induced in B10.RIII mice (Gardner et al., 2015).

Using the flow cytometric gating strategy described above, tissue-resident retinal microglia in the eye were characterized reproducibly as the CD11b^+^NK1.1^neg^Ly6G^neg^Ly6C^low/neg^ population, which stays relatively constant at day 3 post laser-induced CNV (in both retina and RPE-choroid) (Fig. 5). In contrast, we observed a reduction in EIU and a 5 fold increase (from a mean of 2500 to 12,600) in the absolute number of the CD11b^+^NK1.1^neg^Ly6G^neg^Ly6C^low/neg^ cell population of that observed in naïve mice at peak disease EAU (Fig. 5). It is unclear whether this elevation represents the differentiation of infiltrating Ly6C^high^ inflammatory monocytes into Ly6C^low/neg^ macrophages or the proliferation of resident microglia; however, this increase in this population is consistent with an increase in CX_3_CR1^+^ (patrolling monocytes and resident macrophages) population previously reported in EAU (London et al., 2013).

### 3.14 Application and validation of the flow cytometry gating strategy in laser-induced CNV in CCR2^−/−^ animals

Differential expression of the C-C chemokine receptor 2 (CCR2) has been shown between two functional subsets of murine blood monocytes. The so-called ‘inflammatory’ CCR2^+^CX_3_CR1^low^Gr1(Ly6C/G)^+^ subset are actively recruited into inflamed tissues while the ‘patrolling’ CCR2^−^CX_3_CR1^high^Gr1(Ly6C/G)^neg^ subset is recruited to non-inflamed tissues (Geissmann et al., 2003). Signaling through CCR2 facilitates bone marrow egress of myeloid cells and transgenic targeted deletion of CCR2 in mice results in deficient infiltration of myeloid cells and an attenuation in the size of lesions following laser-induced CNV (Tsutsumi et al., 2003).

**Fig. 6:**
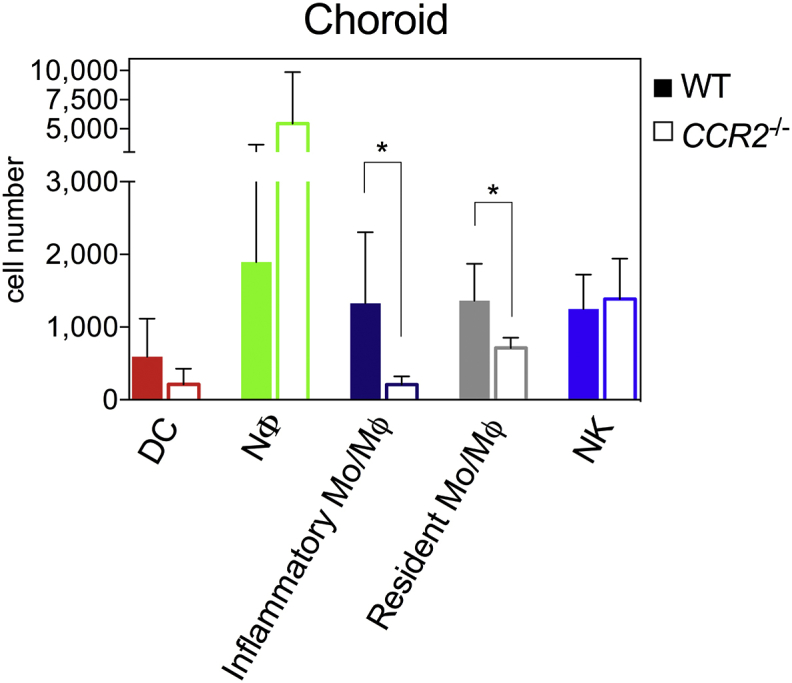
**The myeloid gating strategy confirms diminished inflammatory monocyte infiltration in CCR2 deficient mice during CNV**. Quantitation of absolute numbers of myeloid cells present in day 3 RPE-choroid, post laser-induction of CNV in *Ccr2*^*−/−*^ animals and wild type (WT) controls; n = 6 animals (pooled eyes) per group, mean ± SD; * = P ≤ 0.05 Mann-Whitney (two-tailed); 2 independent experiments; colors in individual bars match individual population gate from Fig. 1, Fig. 4. Although not myeloid cells, CD11b^+^ NK cells are included as they constitute a significant proportion.

To validate the use of the flow cytometry gating strategy under inflammatory conditions and to characterize the reduced population of infiltrating myeloid cells we replicated laser-induced CNV in *Ccr2*^*−/−*^ and wild type controls. Analysis at 3 days post-laser induction revealed a significant reduction in the absolute cell numbers of the inflammatory monocyte/macrophage (CD11b^+^NK1.1^neg^Ly6G^neg^Ly6C^high^) subset in the RPE-choroid of *Ccr2*^*−/−*^ animals as compared to wild type controls (Fig. 6). This is consistent with the decrease in the circulating Ly6C^high^ monocytes seen in *Ccr2*^*−/−*^ animals (Serbina and Pamer, 2006). There was also a significant decrease in the CD11b^+^NK1.1^neg^Ly6G^neg^Ly6C^low-neg^ monocyte/macrophage population in *Ccr2*^*−/−*^ RPE-choroid (Fig. 6). A similar reduction has been documented in circulating Ly6C^low^ blood monocytes in *Ccr2*^*−/−*^ mice, raising the possibility that this CD11b^+^NK1.1^neg^Ly6G^neg^Ly6C^low-neg^ population is affected by the *Ccr2*^*−/−*^ phenotype, by either recruitment or maturation from Ly6C^high^ monocytes (Gutierrez et al., 2011).

## 4 Potential pitfalls and troubleshooting

### 4.1 Resolving myeloid populations in the ocular compartments

Flow cytometric analysis of the ocular compartments is challenging as myeloid cells represent a very small proportion of the retina in steady state. Under inflammatory conditions and leukocyte infiltration the myeloid cell proportion of total retinal cells increases but, as we show in laser-induced CNV, can remain very small. The other non-myeloid cells of the retina represent ‘background noise’ when analyzing myeloid cell populations. Subtle adjustment to increase the forward scatter (FSC; measurement of relative size) detector sensitivity (voltage) can help to improve the resolution of myeloid cells from the differently sized retinal and RPE-choroidal cells, however this may depend on the quality of cellular preparation.

Poor retinal dissociation can result in cellular debris that can impair resolution of myeloid cells. In this case increasing in the FSC threshold may aid in cellular identification; events with a FSC below the threshold become undetectable (generally electronic noise and cellular debris), enabling improved identification of whole cells. When increasing the FSC threshold, careful monitoring of the CD11b^+^ population percentage will ensure that these cells do not fall below the threshold. Addition of an anti-CD45 antibody to the panel may improve resolution of the myeloid cells by excluding the CD45^neg^ non-leukocytes as part of the gating strategy.

When identifying subsets, it is important to use fluorescence-minus controls for each individual experiment to define positive populations. The use of isotype controls has well-described limitations which include variability in staining and failing to account for fluorescence spillover. Due to the multicolor nature of this strategy, we used fluorescence-minus-one controls as for gating, which are currently accepted as the gold standard in flow cytometry (Maecker and Trotter, 2006).

Distinguishing retinal microglia from macrophages is difficult due to several shared antigens, particularly when activated (Guillemin, 2003). Recently, the retinal microglia from *Cx3Cr1*-reporter mice have been characterized using flow cytometry. In keeping with the strategy described here, retinal microglia were identified as CD11b^+^CD11c^low^Ly6G^neg^Ly6C^neg^ (O'Koren et al., 2016).

### 4.2 Autofluorescence

Cellular auto fluorescence poses a potential issue as both retinal and RPE-choroidal tissues have higher intrinsic cellular auto fluorescence than other peripheral tissues (Krause et al., 2014). In laser-induced CNV, this is compounded by higher numbers of dead auto fluorescent cells from tissue damage resulting from laser injury. The use of a live/dead dye allows removal of dead cells from subsequent analysis. Care should be taken when choosing antibody-fluorophore combinations, by avoiding identification of rare populations or low expressed antigens using detectors (PMTs) where auto fluorescence can reduce resolution sensitivity of specific cellular populations, such as the 530/30 detector excited by the 488 nm laser.

### 4.3 F4/80 macrophage marker and the inability to further subdivide the monocyte/macrophage subset

**Fig. 7:**
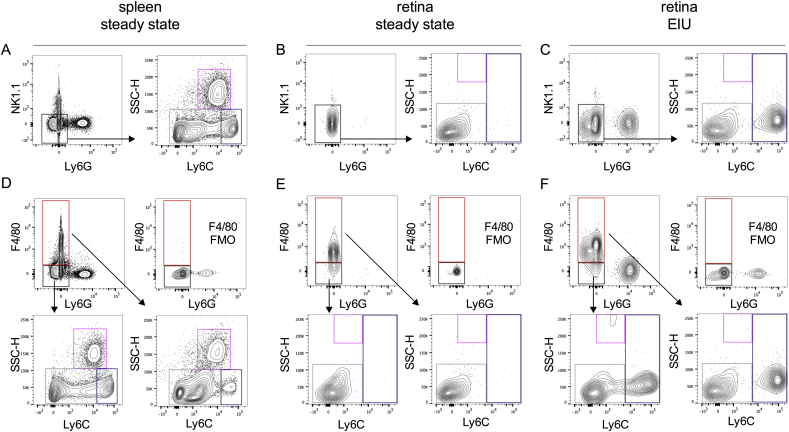
**F4/80 is redundant in defining ‘inflammatory’ Ly6C**^**high**^**and ‘resident’ Ly6C**^**low**^**monocyte/macrophage populations**. Monocytes/macrophages can be divided into Ly6C^high^ and Ly6C^low^ populations in steady state spleen (A), retina (B) and retina following EIU induction (C). To validate whether F4/80 can be used to define inflammatory or resident monocyte/macrophage populations, we repeated the gating strategy using F4/80 instead of NK1.1 to define the monocyte/macrophage population. F4/80 fluorescence-minus-one staining (FMO)s were used to define the F4/80^+^ monocyte/macrophage population in steady state spleen (D), retina (E) and retina following EIU induction. In each tissue, F4/80 did not allow discrimination between ‘inflammatory’ and ‘resident’ populations, with individual F4/80^+^ populations having similar Ly6C staining characteristics to corresponding F4/80^-^ populations; n = 6 animals (pooled eyes); 2 independent experiments.

The F4/80 antigen is commonly used as a marker for macrophages. In their report, Rose et al. omitted the use of an F4/80 antibody in the splenic myeloid compartment as they found that individual F4/80 antibody clones differed in their ability to stain macrophage populations. Moreover, the F4/80 antigen appeared dispensable in defining myeloid subsets in the mouse spleen (Rose et al., 2011). Owing to its redundancy in this gating strategy (Fig. 7), we chose not to incorporate F4/80 into the analysis of myeloid cells in the ocular compartments.

In the spleen, CD11b^+^F4/80^neg^ and CD11b^+^F4/80^+^ populations were analysed for CX_3_CR1 (‘patrolling’ monocyte and resident macrophages) expression (Geissmann et al., 2003). CX_3_CR1 expression was found to be higher in the CD11b^+^F4/80^+^ fraction in comparison to the CD11b^+^F4/80^neg^ compartment. However, no differences in expression of CD115 (blood monocyte) and MHCII (antigen presentation) surface markers were noted between these populations (Breslin et al., 2013, Rose et al., 2011). This raises concerns regarding the level of discrimination between monocytes and macrophages provided by F4/80.

Despite these issues with F4/80, we recognize that both F4/80 and MHC class II have been used to define both macrophages and retinal microglia. As such, individual researchers may choose to add these markers to this strategy as a means of comparing results from this gating system to those previously used.

### 4.4 Discussion

To date, a standardized flow cytometric analysis to characterize ocular myeloid cell populations has been lacking. Using a gating strategy reliably proven for the splenic myeloid compartment proposed by Rose et al., we propose an approach for analyzing myeloid cell populations in the retina and RPE-choroid in several commonly used models of ocular inflammation. This gating strategy is robust and reproducible, even with the use of different fluorophore combinations to those previously reported and provides cytological confirmation that myeloid cell populations are accurately identified, including retinal microglia.

Flow cytometry provides a powerful way to analyse cellular kinetics and dynamics in tissues. This is of particular relevance to the eye during inflammation where it is important to quantify and profile leukocyte infiltration accurately. We demonstrate that this gating strategy highlights phenotypic differences in myeloid cell populations across different inflammatory models, in laser-induced CNV in the *Ccr2*^*−/−*^ deficient mice we demonstrate a significant reduction in numbers of the ‘inflammatory’ CD11b^+^NK1.1^neg^Ly6G^neg^Ly6C^high^ population as well as the CD11b^+^NK1.1^neg^Ly6G^neg^Ly6C^low-neg^ monocyte/macrophage subset.

The flow cytometry gating strategy validated here will aid in the evaluation of the kinetics of infiltrating myeloid cells and their functional role in ocular inflammation. This strategy utilizes 6 antibody-fluorophore combinations, allowing expansion through the addition of antibody/cell surface markers for further phenotyping of each subset identified dependent on both the individual researcher's needs and flow cytometer's capabilities.

## Competing interests

The author U.F.O. Luhmann is employee of F. Hoffmann-La Roche. Ltd. All authors have no conflicts of interest to disclose relating to this work.

## References

[bib1] Ajami B., Bennett J.L., Krieger C., McNagny K.M., Rossi F.M.V. (2011). Infiltrating monocytes trigger EAE progression, but do not contribute to the resident microglia pool. Nat. Neurosci..

[bib2] Auffray C., Sieweke M.H., Geissmann F. (2009). Blood monocytes: development, heterogeneity, and relationship with dendritic cells. Annu. Rev. Immunol..

[bib3] Bainbridge J. (2007). The macrophage is key to choroidal neovascularization in age-related macular degeneration. Ex. Rev. Op..

[bib4] Balaggan K.S., Binley K., Esapa M., MacLaren R.E., Iqball S., Duran Y., Pearson R.A., Kan O., Barker S.E., Smith A.J., Bainbridge J.W.B., Naylor S., Ali R.R. (2006). EIAV vector-mediated delivery of endostatin or angiostatin inhibits angiogenesis and vascular hyperpermeability in experimental CNV. Gene Ther..

[bib5] Breslin W.L., Strohacker K., Carpenter K.C., Haviland D.L., McFarlin B.K. (2013). Mouse blood monocytes: standardizing their identification and analysis using CD115. J. Immunol. Methods.

[bib6] Chen M., Xu H. (2015). Parainflammation, chronic inflammation, and age-related macular degeneration. J. Leukoc. Biol..

[bib7] Chu C.J., Herrmann P., Carvalho L.S., Liyanage S.E., Bainbridge J.W.B., Ali R.R., Dick A.D., Luhmann U.F.O. (2013). Assessment and in vivo scoring of murine experimental autoimmune uveoretinitis using optical coherence tomography. PLoS One.

[bib8] Cortes L.M., Mattapallil M.J., Silver P.B., Donoso L.A., Liou G.I., Zhu W., Chan C.-C., Caspi R.R. (2008). Repertoire analysis and new pathogenic epitopes of IRBP in C57BL/6 (H-2b) and B10.RIII (H-2r) mice. Investig. Ophthalmol. Vis. Sci..

[bib9] Dick A.D. (2003). Control of myeloid activity during retinal inflammation. J. Leukoc. Biol..

[bib10] Gardner P.J., Yazid S., Chu C.J., Copland D.A., Adamson P., Dick A.D., Calder V.L. (2015). TNFα regulates SIRT1 cleavage during ocular autoimmune disease. Am. J. Pathol..

[bib11] Gautier E.L., Shay T., Miller J., Greter M., Jakubzick C., Ivanov S., Helft J., Chow A., Elpek K.G., Gordonov S., Mazloom A.R., Ma'ayan A., Chua W.-J., Hansen T.H., Turley S.J., Merad M., Randolph G.J., Gautier E.L., Jakubzick C., Randolph G.J., Best A.J., Knell J., Goldrath A., Miller J., Brown B., Merad M., Jojic V., Koller D., Cohen N., Brennan P., Brenner M., Shay T., Regev A., Fletcher A., Elpek K., Bellemare-Pelletier A., Malhotra D., Turley S., Jianu R., Laidlaw D., Collins J., Narayan K., Sylvia K., Kang J., Gazit R., Garrison B.S., Rossi D.J., Kim F., Rao T.N., Wagers A., Shinton S.A., Hardy R.R., Monach P., Bezman N.A., Sun J.C., Kim C.C., Lanier L.L., Heng T., Kreslavsky T., Painter M., Ericson J., Davis S., Mathis D., Benoist C. (2012). Gene-expression profiles and transcriptional regulatory pathways that underlie the identity and diversity of mouse tissue macrophages. Nat. Immunol..

[bib12] Geissmann F., Jung S., Littman D.R. (2003). Blood monocytes consist of two principal subsets with distinct migratory properties. Immunity.

[bib13] Geissmann F., Manz M.G., Jung S., Sieweke M.H., Merad M., Ley K. (2010). Development of monocytes, macrophages, and dendritic cells. Science.

[bib14] Gordon S., Plüddemann A., Martinez Estrada F. (2014). Macrophage heterogeneity in tissues: phenotypic diversity and functions. Immunol. Rev..

[bib15] Guillemin G.J. (2003). Microglia, macrophages, perivascular macrophages, and pericytes: a review of function and identification. J. Leukoc. Biol..

[bib16] Gutierrez D.A., Kennedy A., Orr J.S., Anderson E.K. (2011). Aberrant accumulation of undifferentiated myeloid cells in the adipose tissue of CCR2-deficient mice delays improvements in insulin sensitivity. Diabetes.

[bib17] Hashimoto D., Chow A., Noizat C., Teo P., Beasley M.B., Leboeuf M., Becker C.D., See P., Price J., Lucas D., Greter M., Mortha A., Boyer S.W., Forsberg E.C., Tanaka M., van Rooijen N., García-Sastre A., Stanley E.R., Ginhoux F., Frenette P.S., Merad M. (2013). Tissue-resident macrophages self-maintain locally throughout adult life with minimal contribution from circulating monocytes. Immunity.

[bib18] Hume D.A. (2011). Applications of myeloid-specific promoters in transgenic mice support in vivo imaging and functional genomics but do not support the concept of distinct macrophage and dendritic cell lineages or roles in immunity. J. Leukoc. Biol..

[bib19] Imai Y., Ibata I., Ito D., Ohsawa K., Kohsaka S. (1996). A novel gene iba1 in the major histocompatibility complex class III region encoding an EF hand protein expressed in a monocytic lineage. Biochem. Biophys. Res. Commun..

[bib20] Janeway C.A., Medzhitov R. (2002). Innate immune recognition. Annu. Rev. Immunol..

[bib21] Jutila M.A., Kroese F.G., Jutila K.L., Stall A.M., Fiering S., Herzenberg L.A., Berg E.L., Butcher E.C. (1988). Ly-6C is a monocyte/macrophage and endothelial cell differentiation antigen regulated by interferon-gamma. Eur. J. Immunol..

[bib22] Kerr E.C., Raveney Ben J.E., Copland D.A., Dick A.D., Nicholson L.B. (2008). Analysis of retinal cellular infiltrate in experimental autoimmune uveoretinitis reveals multiple regulatory cell populations. J. Autoimmun..

[bib23] Kettenmann H., Hanisch U.K., Noda M., Verkhratsky A. (2011). Physiology of microglia. Physiol. Rev..

[bib24] Kezic J., Xu H., Chinnery H.R., Murphy C.C., McMenamin P.G. (2008). Retinal microglia and uveal tract dendritic cells and macrophages are not CX3CR1 dependent in their recruitment and distribution in the young mouse eye. Investig. Ophthalmol. Vis. Sci..

[bib25] Krause T.A., Alex A.F., Engel D.R., Kurts C., Eter N. (2014). Vegf-production by CCR2-dependent macrophages contributes to laser-induced choroidal neovascularization. PLoS One.

[bib26] London A., Benhar I., Mattapallil M.J., Mack M., Caspi R.R., Schwartz M. (2013). Functional macrophage heterogeneity in a mouse model of autoimmune central nervous system pathology. J. Immunol..

[bib27] Luhmann U.F.O., Ali R.R. (2011). Local Vs. Systemic Mononuclear Phagocytes in Age-related Macular Degeneration and Their Regulation by *CCL2–CCR2* and *CX3CL1–CX3CR1* Chemokine Signalling.

[bib28] Luhmann U.F.O., Lange C.A., Robbie S., Munro P.M.G., Cowing J.A., Armer H.E.J., Luong V., Carvalho L.S., MacLaren R.E., Fitzke F.W., Bainbridge J.W.B., Ali R.R. (2012). Differential modulation of retinal degeneration by Ccl2 and Cx3cr1 chemokine signalling. PLoS One.

[bib29] Maecker H.T., Trotter J. (2006). Flow cytometry controls, instrument setup, and the determination of positivity. Cytom. A.

[bib30] Murray P.J., Allen J.E., Biswas S.K., Fisher E.A., Gilroy D.W., Goerdt S., Gordon S., Hamilton J.A., Ivashkiv L.B., Lawrence T., Locati M., Mantovani A., Martinez F.O., Mege J.-L., Mosser D.M., Natoli G., Saeij J.P., Schultze J.L., Shirey K.A., Sica A., Suttles J., Udalova I., Van Ginderachter J.A., Vogel S.N., Wynn T.A. (2014). Perspective. Immunity.

[bib31] Murray P.J., Wynn T.A. (2011). Protective and pathogenic functions of macrophage subsets. Nat. Rev. Immunol..

[bib32] Newson J., Stables M., Karra E., Arce-Vargas F., Quezada S., Motwani M., Mack M., Yona S., Audzevich T., Gilroy D.W. (2014). Resolution of acute inflammation bridges the gap between innate and adaptive immunity. Blood.

[bib33] O'Koren E.G., Mathew R., Saban D.R. (2016). Fate mapping reveals that microglia and recruited monocyte-derived macrophages are definitively distinguishable by phenotype in the retina. Sci. Rep..

[bib34] Patel A.R., Ritzel R., McCullough L.D., Liu F. (2013). Microglia and ischemic stroke: a double-edged sword. Int. J. Physiol. Pathophysiol. Pharmacol..

[bib35] Perfetto S.P., Chattopadhyay P.K., Roederer M. (2004). Seventeen-colour flow cytometry: unravelling the immune system. Nat. Rev. Immunol..

[bib36] Ponomarev E.D., Shriver L.P., Maresz K., Dittel B.N. (2005). Microglial cell activation and proliferation precedes the onset of CNS autoimmunity. J. Neurosci. Res..

[bib37] Rose S., Misharin A., Perlman H. (2011). A novel Ly6C/Ly6G-based strategy to analyze the mouse splenic myeloid compartment. Cytometry.

[bib38] Sakurai E., Anand A., Ambati B.K., van Rooijen N., Ambati J. (2003). Macrophage depletion inhibits experimental choroidal neovascularization. Investig. Ophthalmol. Vis. Sci..

[bib39] Schulz C., Perdiguero E.G., Chorro L., Szabo-Rogers H., Cagnard N., Kierdorf K., Prinz M., Wu B., Jacobsen S.E.W., Pollard J.W., Frampton J., Liu K.J., Geissmann F. (2012). A lineage of myeloid cells independent of myb and hematopoietic stem cells. Science.

[bib40] Serbina N.V., Pamer E.G. (2006). Monocyte emigration from bone marrow during bacterial infection requires signals mediated by chemokine receptor CCR2. Nat. Immunol..

[bib41] Trittibach P., Barker S.E., Broderick C.A., Natkunarajah M., Duran Y., Robbie S.J., Bainbridge J.W.B., Smith A.J., Sarra G.-M., Dick A.D., Ali R.R. (2008). Lentiviral-vector-mediated expression of murine IL-1 receptor antagonist or IL-10 reduces the severity of endotoxin-induced uveitis. Gene Ther..

[bib42] Tsutsumi C., Sonoda K.-H., Egashira K., Qiao H., Hisatomi T., Nakao S., Ishibashi M., Charo I.F., Sakamoto T., Murata T., Ishibashi T. (2003). The critical role of ocular-infiltrating macrophages in the development of choroidal neovascularization. J. Leukoc. Biol..

[bib43] Vivier E., Tomasello E., Baratin M., Walzer T., Ugolini S. (2008). Functions of natural killer cells. Nat. Immunol..

[bib44] Yu J., Freud A.G., Caligiuri M.A. (2013). Location and cellular stages of natural killer cell development. Trends Immunol..

[bib45] Zhang G.X., Li J., Ventura E., Rostami A. (2002). Parenchymal microglia of naïve adult C57BL/6J mice express high levels of B7.1, B7.2, and MHC class II. Exp. Mol. Pathol..

